# Brucellosis, Taiwan, 2011

**DOI:** 10.3201/eid1712.110739

**Published:** 2011-12

**Authors:** Yu-Chung Chuang, Szu-Chi Chen, Jung-Jung Mu, Hsiu-Ying Lin, Chih-Hsin Chang, Wei-Shiung Yang, Po-Ren Hsueh

**Affiliations:** National Taiwan University Hospital, Taipei, Taiwan (Y.-C. Chuang, S.-C. Chen, H.-Y. Lin, C.-H. Chang, W.-S. Yang, P.-R. Hsueh);; Centers for Disease Control, Taipei (J.-J. Mu)

**Keywords:** Brucellosis, Taiwan, zoonoses, bacteria

**To the Editor:** Human brucellosis is the most common zoonosis worldwide (*1**–**4*). The disease is transmitted to humans through the consumption of infected meat and raw dairy products from domestic livestock or by direct or indirect contact with infected animals (*1**–**3*). The disease is multisystemic and shows wide clinical polymorphism (*2**–**4*).

A 54-year-old woman reported high fever, poor appetite, epigastralgia, mild dysuria, generalized myalgia, and mild left side pain for 6 days before she sought care at and was admitted to National Taiwan University Hospital, Taipei, Taiwan. She had a history of ovarian cancer (clear cell, stage Ic), which had been treated with surgery and chemotherapy 7 years earlier at our hospital. She had traveled to many countries, most recently to Algeria and Morocco 2 months before this admission. During her stay in North Africa, she had close contact with camels, ate cheese and yogurt, and drank milk, even in the desert. Fever occurred 1 month after she returned to Taiwan.

On physical examination, her body temperature was 39.9°C, blood pressure was 97/68 mm Hg, and pulse rate was 89 beats/min. There was mild tenderness on palpation in the epigastric area. Laboratory analysis of serum specimens showed elevated levels of alanine aminotransferase (534 U/L), aspartate aminotransferase (841 U/L), and alkaline phosphatase (337 U/L) but a total bilirubin level (0.48 mg/dL) within reference limits. Renal function was within reference ranges (blood urea nitrogen 9.5 mg/dL, creatinine 0.6 mg/dL). C-reactive protein was elevated (4.59 mg/dL), but procalcitonin level was within reference range (0.13 ng/mL). The leukocyte count was 4,710 cells/mm^3^, and hemoglobin was 11.4 g/dL. Serologic tests for viral hepatitis were negative for hepatitis B virus, hepatitis A virus, cytomegalovirus, and Epstein-Barr virus infections. Abdominal ultrasound indicated mild splenomegaly and no evidence of vegetation. Abdominal and pelvic computed tomography showed focal splenic infarction with splenomegaly.

Empirical ceftriaxone (1 g every 12 h) and doxycycline (100 mg every 12 h) were administered, and fever subsided 5 days later. Two aerobic culture bottles (BacT/ALERT, bioMérieux Inc., La Balme les Grottes, France) from different sets of blood cultures on the day before admission yielded unidentified gram-negative tiny bacilli after 2 days of incubation. The organism was identified as *Brucella melitensis* by the Vitek 2 GN identification system (bioMérieux Inc.) (probability of identity 99%) and was confirmed by analysis of partial 16S rRNA gene sequencing. Two primers were used: 8FPL (5′-AGAGTTTGATCCTGGCTCAG-3′) and 1492RPL (5′-GGTTACCTTGTTACGACTT-3′). We compared the partial sequences with published sequences in the GenBank database by using the BLASTN algorithm (www.ncbi.nlm.nih.gov/blast). The closest match was *B. melitensis* (GenBank accession no. CP001852.1; maximal identity 100%). MICs were determined by the Etest (AB Biodisk, Solna, Sweden) on Mueller-Hinton agar (BBL, Becton Dickinson, Sparks, MD, USA) supplemented with 5% sheep blood and were interpreted 2 days after incubation. The isolate was susceptible to doxycycline (MIC 0.25 μg/mL; susceptible MICs <2 μg/mL) but not susceptible to trimethoprim/sulfamethoxazole (MIC 1/19 μg/mL; susceptible MICs <0.5 μg/mL) (*5**,**6*). MIC values of tigecycline and gentamicin were 0.125 μg/mL and 2.0 μg/mL, respectively. A serum sample for examination of *Brucella* antibody by Rose Bengal test using *B. abortus* antigen (VLA Scientific, Winchester, UK) collected 12 days after fever onset was positive.

Antimicrobial drug treatment was changed to doxycycline and gentamicin. However, low-grade fever and low back pain developed 2 days after administration of gentamicin. The back pain was attributed to muscle pain and was almost completely relieved by 2-day treatment with a nonsteroidal antiinflammatory drug. Whole-body gallium scan and spine magnetic resonance imaging suggested osteomyelitis and epidural abscess over the third and fourth lumbar spines. The patient was treated with doxycycline for 6 weeks. A liver function test 2 weeks after admission showed values within reference limits.

Cases of human brucellosis and animal sources of *Brucella* spp. have been reported from Algeria and Morocco (*7**–**10*). The most common laboratory findings in patients with brucellosis are high C-reactive protein levels and anemia (*3**,**4*). This patient had high C-reactive protein levels but procalcitonin values within reference limits at admission. Hepatic involvement of brucellosis has been reported to range from 2% to 25% (*3*). The patient also had acute anicteric hepatitis, and serologic test results were negative for all hepatotropic viruses. The isolate from this patient exhibited high MICs for trimethoprim/sulfamethoxazole, a finding rarely reported (*5**,**6*). The low MIC value of tigecycline suggests the potential role of this agent for the treatment of brucellosis.

This report confirms brucellosis in Taiwan. Brucellosis could become an emerging problem in this country, particularly given the frequency of travel between Taiwan and areas where brucellosis is endemic.
